# Sex differences in response to kanamycin-induced ototoxicity in C57BL/6 J mice

**DOI:** 10.1038/s41598-025-21962-y

**Published:** 2025-10-31

**Authors:** Natalia Smith-Cortinez, Louise V. Straatman, Ferry G. J. Hendriksen, Robert J. Stokroos, Huib Versnel

**Affiliations:** 1 Department of Otorhinolaryngology and Head & Neck Surgery, University Medical Center Utrecht, Utrecht, the Netherlands; 2 UMC Utrecht Brain Center, University Medical Center Utrecht, Utrecht University, Utrecht, the Netherlands

**Keywords:** Ototoxicity, Hearing loss, Sex differences, Kanamycin, C57BL/6 J mice, Auditory system, Neuroscience

## Abstract

Sensorineural hearing loss (SNHL) is caused mainly by irreversible damage to sensory hair cells after noise exposure, ototoxic medication, and ageing, with men being more prone to developing SNHL than women are. For animal models, sex-related susceptibility to develop SNHL needs to be taken into account. Ototoxic trauma can be modeled in mice via the systemic administration of furosemide and kanamycin. Several other deafening models have shown differences in response to ototoxic medication between female and male mice. Differences in response to kanamycin-induced ototoxicity in male and female mice have not been studied. Here, we examined sex differences in susceptibility to kanamycin plus furosemide-induced ototoxicity in adult C57BL/6 J mice. Adult (postnatal day 40) female and male C57BL/6 J and Lgr5GFP (C57BL/6 J background) mice were used. The animals were deafened with a single dose of furosemide (100 mg/kg, i.v.) in combination with kanamycin (700 or 900 mg/kg, s.c.). Before deafening, seven and twenty-eight days after deafening, auditory brainstem responses (ABRs) to click stimuli were recorded to evaluate hearing performance. The cochleae were harvested seven or twenty-eight days after the induction of ototoxicity and processed for histology to evaluate hair cell loss. Male mice presented large ABR threshold shifts after ototoxic treatment with 700 mg/kg kanamycin (50 dB median hearing loss). In males, 9/12 mice had threshold shifts greater than 40 dB. However, female mice presented significantly less hearing loss in response to 700 mg/kg kanamycin, as observed via ABRs after ototoxic treatment (32 dB median hearing loss), and only 8/22 female mice presented threshold shifts greater than 40 dB. When treated with 900 mg/kg kanamycin, female mice presented large ABR threshold shifts after ototoxic treatment (50 dB median), and 11/16 mice presented threshold shifts greater than 40 dB. Female C57BL/6 J mice are less susceptible to kanamycin-induced hearing loss than males are and hence need higher doses of kanamycin to reach the same level of hearing loss. This finding is in line with the prevalence of disabling hearing loss in humans, which is 7.3% in males and 4.8% in females, where estrogens have been linked to increased hearing performance and protection against hearing loss.

**Supplementary Information:**

The online version contains supplementary material available at 10.1038/s41598-025-21962-y.

## Introduction

Hearing loss is the most frequent sensory deficit in humans and occurs when cochlear sensory hair cells (HCs) and/or their associated neurons (spiral ganglion neurons, SGNs) are damaged. Irreversible HC loss is particularly caused by aging, noise exposure and ototoxic medication. In 2021, there were approximately 430 million individuals with disabling hearing loss, and according to the World Health Organization (WHO), this number might increase to more than 700 million individuals by 2050, requiring rehabilitation^1^. Hearing loss not only affects communication and spatial navigation but it also is associated with tinnitus; it results in high levels of morbidity, depression, and social isolation and is known to contribute to cognitive decline in elderly individuals^2–4^. The prevalence of hearing loss is lower in women than in men, and the onset of hearing loss is later in women^5–12^. This sex dependence has also been shown specifically for noise-induced hearing loss^13–17^. Lower levels of estrogens have been associated with greater hearing vulnerability in postmenopausal women^18–20^, and indeed, estrogens have been shown to protect against noise-induced hearing loss in animal experiments^21^.

In hearing research, animal models are used to examine mechanisms of damage (disease), evaluate new otoprotective medications (drug development), and find novel therapies that promote the survival or regeneration of sensory HCs and SGNs. Historically, only male subjects have been used in animal studies^22^. This is mostly due to the misbelief that female research animals are intrinsically more variable than males are, which is too troublesome for routine inclusion in research protocols and due to the difficulties in planning experiments around the female cycle. This means that therapies or mechanisms fail to translate directly to the human situation because of a lack of inclusion of females. This implies a disadvantage for women versus men since therapies are not tailored to them. The sex bias in basic and preclinical hearing loss research can lead to a less comprehensive understanding of sex-based mechanistic differences and prevent the development of robust treatment options for all patients^23^. Among the studies evaluating noise-induced hearing loss published between 2011 and 2015, only 73% reported the sex of the animals used; of those studies, 61% used males only, and 14% used females only^24^. Among the total studies included that reported sex and the number of animals used, 67% of the animals were males, and 33% were females^24^.

Ototoxic trauma can be modeled in mice with a systemic administration of furosemide and kanamycin^25^, but the effects of different doses of kanamycin have been reported only in male subjects. Although sex differences in the response to age-related and noise-induced hearing loss in mouse models have been previously reported^26–29^, to our knowledge, only one study has reported such differences in ototoxicity^30^. This study by DeBacker et al. revealed that male BALB mice are more sensitive to cisplatin-induced hearing loss than females are and that, in contrast, C57BL/6 J female mice are more sensitive to the same treatment than males are^30^. The responses of males and females to kanamycin-induced ototoxicity have not yet been compared. To avoid underrepresentation of female mice in animal experiments using kanamycin-induced ototoxicity and from the perspective of the 3Rs principles, allowing the use of both male and female mice in animal experimentation, we included females in a preclinical study from our group on inner ear regeneration using adult Lgr5-EGFP-IRES-creERT2 (Lgr5GFP) transgenic (C57BL/6 J background) mice^31^. Compared with males, females were less sensitive (more resilient) to ototoxic medication. Hence, we examined sex differences in susceptibility to kanamycin plus furosemide-induced ototoxicity in an additional group of adult C57BL/6 J mice.

## Methods

### Animals

We used 4–6-week-old C57BL/6 J (cohort 1) and re-used data from Lgr5GFP transgenic (C57BL/6 J background, cohort 2) male and female mice used in a previous study^31^. Mice were bought from The Jackson Lab and bred in the Central Laboratory Animal Research Facility of the UMC Utrecht and University of Utrecht. Ten male and 20 female C57BL/6 J mice were used, and 3 male and 18 female Lgr5GFP transgenic mice were used. Same sex litter mates were housed together in individually ventilated cages with two or four mice per cage with food and water ad libitum and standard laboratory conditions. The breeding, surgical and experimental procedures were approved by/ and performed in accordance with the guidelines and regulations by the Dutch Central Authority for Scientific Procedures on Animals (CCD:1,150,020,186,105). All experimental procedures are reported in accordance with ARRIVE guidelines.

### Deafening procedure

Male and female mice were deafened under isoflurane anesthesia and on a heated pad by the subcutaneous injection of kanamycin sulfate at a dosage of 700 mg/kg to male mice (M700) and 700 or 900 mg/kg to female mice (F700 or F900, respectively) in 100 mg/ml stock solution in saline. To reduce the number of animals no normal hearing animals were used in this study as control. Also, because our aim was 1. to investigate the sex differences on the standard kanamycin dose and 2. to evaluate if female mice need higher doses of kanamycin to reach hearing loss, we did not include a M900 group. Five minutes after kanamycin injection 100 mg/kg furosemide was given by a tail vein injection of (stock solution 100 mg/ml). The mice were allowed to recover in a heated pad before being returned to the cage. Deafened mice were followed for 7 and/or 28 days after ototoxicity. The mice were weighed before the deafening procedure and weekly after the deafening procedure as part of welfare monitoring. On day 7 (for cohort 2) or day 28 (for cohort 1) of the animal experiment, the mice were killed by CO_2_ asphyxiation, and the cochleae were harvested after decapitation.

### Auditory brainstem responses (ABRs)

The hearing of the mice was evaluated via click-evoked auditory brainstem responses (ABRs). The mice were anesthetized under isoflurane anesthesia in a soundproof and electrically insulated box while the ABRs were recorded. Subdermal needle electrodes were placed behind the right pinna (active), on the skull (reference), and in the hind limb (ground) via a subdermal approach. Acoustic stimuli clicks (width of 20 µs) were generated and attenuated via a TDT3 system (Multi-I/O processor RZ6; Tucker-Davis Technologies, Alachua, FL, USA). A Bowers & Wilkins speaker (CCM683; 8 Ω; 25—130 W) placed 5 cm from the right ear presented the sound stimuli in the free field with an interstimulus interval of 33 ms. A Princeton Applied Research (Oak Ridge, TN, USA) preamplifier (amplification × 5000; bandpass filter 0.1–10 kHz) was used to preamplify the electrode signals, which were subsequently digitalized for analysis by the same TDT3 system. The responses were averaged over a maximum of 500 repetitions and stored on a computer for offline analysis via custom MATLAB software. The sound level was reduced in 10 dB decrements, starting from the maximum sound level of approximately 105 dB peak equivalent SPL (peSPL), until it was 10 dB below the sound level with no visible ABR response. The threshold was defined as the interpolated sound level at which the amplitude of the largest ABR wave was 0.5 µV peak to peak. The threshold was noted as 105 dB peSPL in case of absent responses (i.e., ABR amplitude < 0.5 µV at 105 dB peSPL).

### Analysis of ABRs

**Fig. 1:**
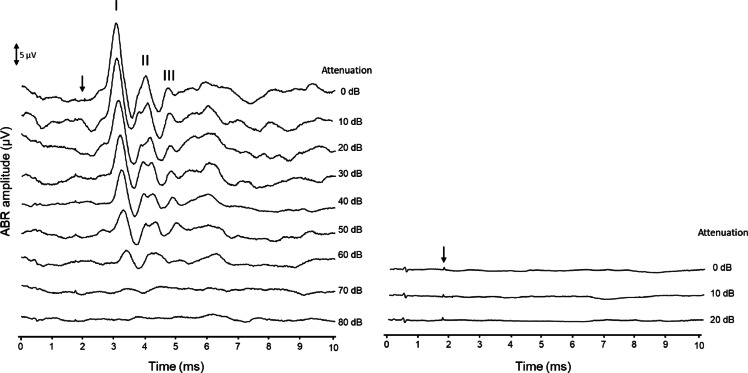
Representative auditory brainstem response (ABR) from one mouse on day 0 before deafening. Representative auditory brainstem responses (ABRs) of one C57BL/6 J mouse before and after deafening. The levels are shown in dB attenuation, with 0 dB being equal to 105 dB peSPL. ABR thresholds before deafening 36 dB peSPL and ABR threshold after deafening 105 dB peSPL. Peaks I (I), II (II) and III were identified and labeled. Arrows indicate the start of the auditory stimuli.

During the ABR analysis, the investigator was blinded to the study population (M700, F700, or F900). The raw ABR traces were visually examined to confirm signal quality and consistency across recordings. Furthermore, the baseline threshold of the mice was examined, and we applied a threshold of ≤ 55 dB peSPL on day 0 as an inclusion criterion for normal hearing for further analysis. This resulted in the exclusion of 1 male mouse. We applied a ≥ 40 dB threshold shift as the criterion for a positive response to ototoxic treatment. If the threshold shift was less than 40 dB, the animal was considered a non-responder. The ≥ 40 dB criterion was chosen as it typically indicates severe (outer) hair cell loss^32^. Figure 1 shows a representative ABR of one mouse from the study on day 0. To calculate the threshold, first, the thresholds were determined for peak I, peak II and peak III via interpolation via a criterion of 0.5 µV, and then the lowest threshold was selected.

### Cryosectioning, whole-mount sample preparation and immunofluorescence microscopy

Mouse cochleae were harvested after termination by CO2 asphyxiation and processed for cryosectioning and whole-mount preparations as described previously^32^. Briefly, after fixation (2% paraformaldehyde, Sigma–Aldrich), the cochleae were decalcified at room temperature for 7 days. Cryopreservation was performed via a sucrose gradient, after which the tissues were embedded in compound (Sakura Finetek Europe B.V., Alphen aan den Rijn, the Netherlands) and stored at − 80 °C. Cryosections of 12 µm were cut using a Leica CM3050 cryostat and mounted on microscope slides. For whole-mount samples, cochleae were fixed the otic capsule was opened, the lateral wall, Reissner’s membrane, tectorial membrane and modiolus were removed, and the basilar membrane carrying the organ of Corti was dissected into individual half-turns. Immunofluorescence staining was performed on cryosections and whole-mount dissections. The tissues and slides were washed with blocking solution (2% donkey serum, 5% fetal calf serum and 0.1%–0.5% Triton X-100 in PBS). The samples were incubated with an anti-myosin VIIA primary antibody (MYO7A, 1/200, rabbit, Proteus Biosciences, 25–6790) overnight at 4 °C. Later, the slides and tissues were washed with blocking solution and incubated with the secondary antibodies donkey-anti-rabbit-Alexa 594 (1/500, Invitrogen, A-21207) and DAPI solution (1/500, Abcam, AB228549) for 90 min at room temperature. Finally, the samples were washed in PBS and mounted in Vectashield Antifade Mounting Medium (Vector laboratories, H-1000). The slides were imaged via a Zeiss LSM700 scanning confocal microscope. Three-dimensional image reconstructions of Z-stacks were performed via ImageJ^33^ 1.47v software (https://imagej.net/).

### Cell counting

The cells were counted by two independent raters via whole-mount dissection immunofluorescence staining images. The total number of IHCs and OHCs was determined by analyzing MYO7A + cells. The density was then calculated for each segment of cells per 100 μm. We evaluated the IHC and OHC counts by assessing the number of HCs alongside the basilar membrane in the base (25% distance from the base), middle (50% distance from the base) and apex (75% distance from the base) in cochlear whole mounts from cohort 1 mice.

### Statistical analysis

This study used SPSS statistics to conduct Mann‒Whitney and chi‒square tests to investigate the differences between the ABR thresholds in M700 and F700 groups and between the F700 and F900 groups. The distribution of threshold shifts is bimodal with on one side small shifts, and on the other large shifts, thus the distribution is not normal; hence we used non-parametric Mann–Whitney tests. The Wilcoxon test was used to analyze within-animal differences in ABR thresholds. A mixed effects model was used to evaluate differences in IHC and OHCs. The statistical analysis indicated a significant difference when the *p* value was less than 0.05. Graphs were generated via GraphPad Prism 10.

## Results

### Baseline ABR thresholds did not differ between female and male mice

**Fig. 2:**
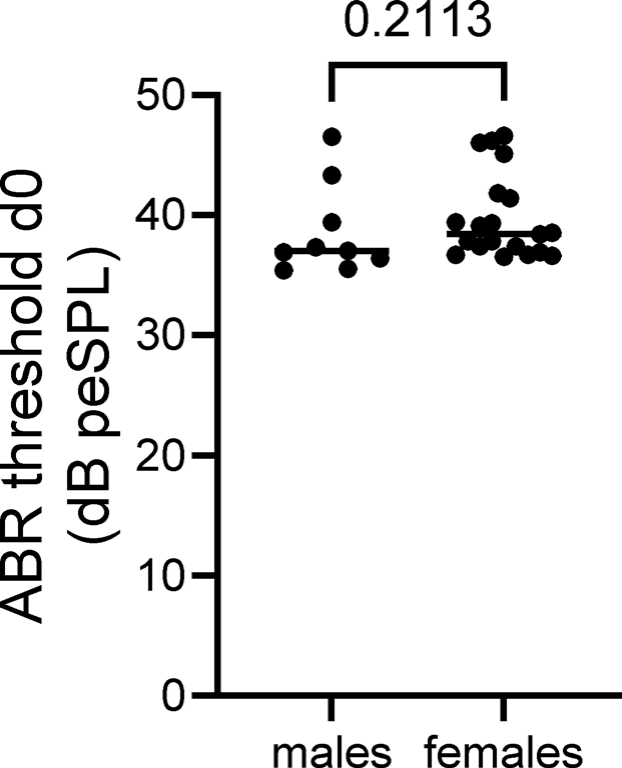
ABR thresholds on day 0. ABR thresholds (in dB peSPL) on day 0 for all male and female mice from cohort 1 (n = 10 males and 20 females).

We analyzed the ABR thresholds of cohort 1 C57BL/6 J mice on day 0 (Supplementary Table 1). Analyses on day 0 were performed independent of the deafening strategy used to compare baseline measurements between females and males. We observed no significant differences in ABR thresholds on day 0 between male and female mice (*p* = 0.21, U = 63, Mann‒Whitney test; Fig. 2).

### No significant differences in ABR thresholds between 7 and 28 days after deafening

**Fig. 3:**
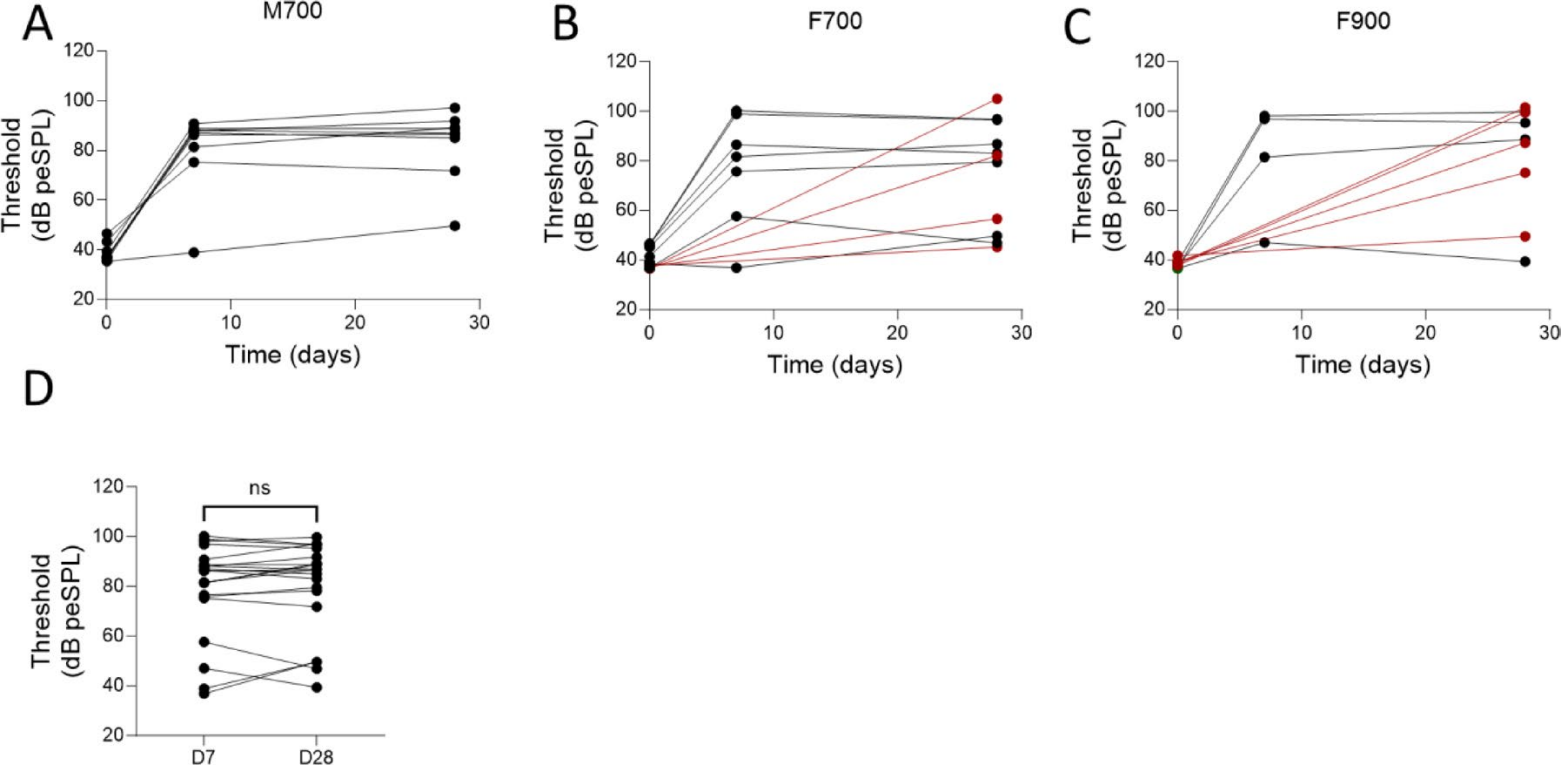
ABR thresholds in male and female mice from cohort 1 treated with 700 or 900 mg/kg kanamycin. ABR thresholds (in dB peSPL) at days 0, 7, and 28 (**A**) for the group of male mice that received a dose of 700 mg/kg kanamycin (M700), n = 9 mice; (**B**) for the group of female mice that received a dose of 700 mg/kg kanamycin (F700) (n = 7 mice measured at days 0, 7 and 28; red dots, n = 4 mice measured at days 0 and 28) In one case we found no responses at the maximum click level, which in the plot is marked as 105 dB peSPL; and (**C**) for the group of female mice that received a dose of 900 mg/kg kanamycin (n = 4 mice measured at days 0, 7 and 28; red dots, n = 6 mice measured at days 0 and 28). (**D**) ABR thresholds at days 7 and 28 of all 20 mice that were measured at both timepoints (n = 9 M700; n = 7 F700; and n = 4 F900).

ABR thresholds for days 0, 7 and 28 after deafening were plotted for the M700 (Fig. 3A), F700 (Fig. 3B), and F900 (Fig. 3C) groups. 8/9 mice presented elevated ABR thresholds at 7 and 28 days after deafening in the group M700 compared to day 0 (Fig. 3A). 3/7 mice presented ABR threshold shifts of > 40 dB at 7 and 28 days after deafening in the group of F700 compared to day 0 (Fig. 3B). For 4 mice in F700, ABRs were missing on day 7; of these, 2 mice showed elevated ABR thresholds 28 days after deafening compared with day 0 (red lines, Fig. 3B**).** We observed in he group F900, 3/4 mice presented elevated ABR thresholds at 7 and 28 days after deafening compared with day 0 (Fig. 3C). For 5 mice in F900, ABRs were missing on day 7; of these, 4/5 mice showed elevated ABR thresholds 28 days after deafening compared with day 0 (red lines, Fig. 3B**).** We observed no significant differences between the ABR thresholds at days 7 and 28 in any of the 21 mice measured at either time points (*p* = 0.35, W = 51, two-tailed Wilcoxon test, Fig. 3D).

### Hair cell loss after ototoxicity in M700, F700 and F900

**Fig. 4:**
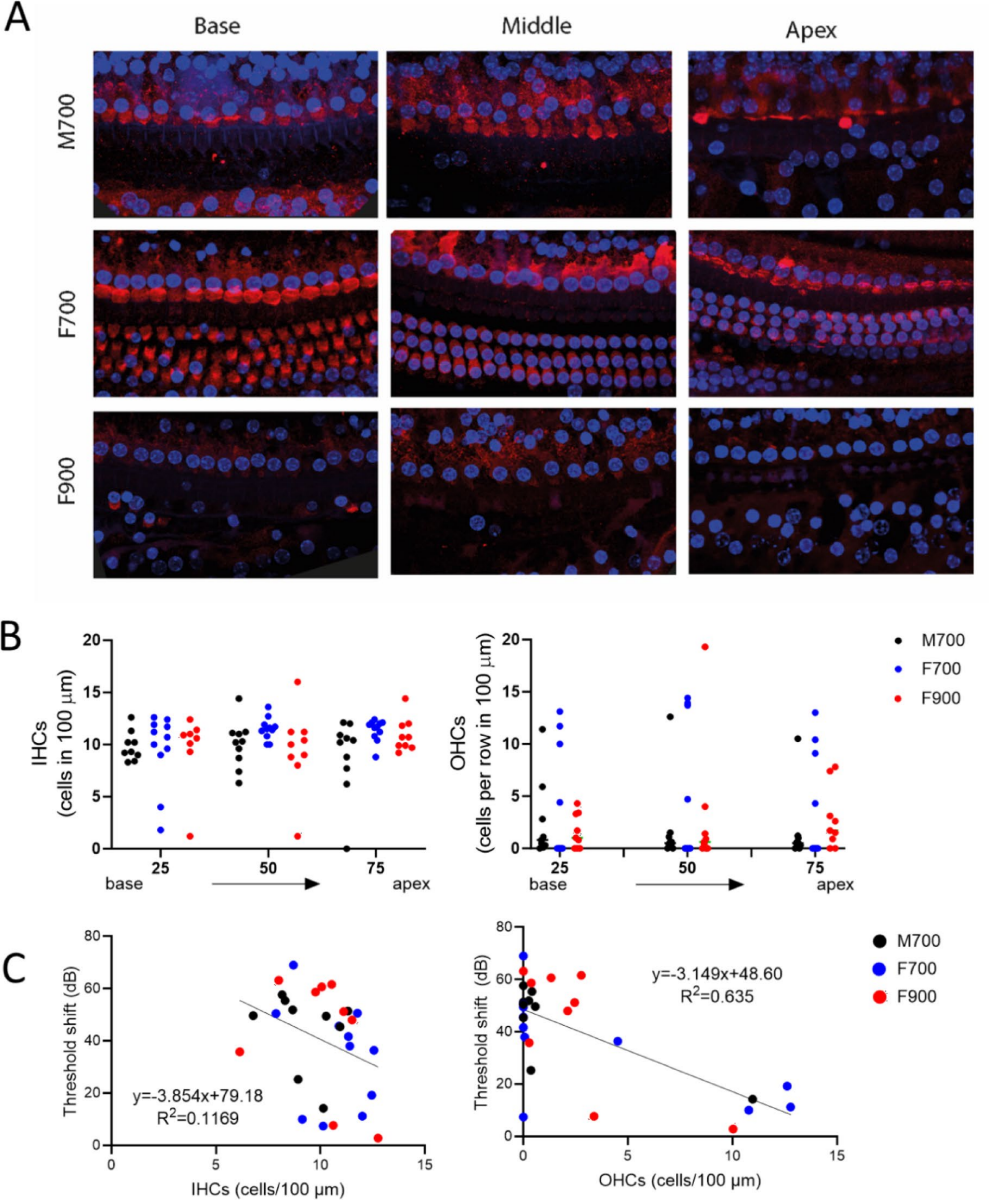
Inner and outer hair cell losses in male and female mice treated with 700 or 900 mg/kg kanamycin. (**A**) Representative images of immunofluorescence microscopy images of the apex, middle and base in whole-mount dissections of the cochleae of male mice that were ototoxically-deafened with 700 mg/kg kanamycin (M700), and of female mice treated with 700 (F700) or 900 (F900) mg/kg kanamycin. Whole mounts were stained with myosin VIIA (MYO7A) in red and with DAPI in blue to visualize inner (IHCs) and outer (OHCs) hair cells. MYO7A staining revealed that the OHCs were completely abolished after deafening in the M700 and F900 examples, with partial preservation of IHCs, and showed survival of most OHCs in the F700 example. (**B**) IHCs (left panel) and OHCs (right panel) counts per row in 100 µm tissue from whole-mount images in apex, middle and base of the cochleae of M700, F700 or F900 mice. (**C**) IHC count versus ABR threshold shift (left panel) and OHC count versus ABR threshold shift (right panel) for all the mice in groups M700, F700 and F900. There was a significant correlation between ABR threshold shifts and the numbers of OHCs but not IHCs. No significant differences were found between M700 and F700 in IHC or OHC counts.

We observed survival of IHCs in most cochlear whole mounts from groups M700, F700 and F900 (Fig. 4A, B). We observed that most cochlear whole mounts had 50% or less surviving OHCs; however, 1 mouse in group M700 and 3 mice in group F700 had survival of most OHCs (Fig. 4B and Supplementary Table 2). Differences in IHC (*p* = 0.118 mixed effects model) or OHC (*p* = 0.377 mixed effects model) counts between M700 and F700 were not significant. We evaluated the correlation of the threshold shifts versus average IHC and OHC counts independent of the groups (linear regression analysis, Fig. 4C and Supplementary Table 2). The threshold significantly increased with decreasing OHC counts (*p* < 0.0001, R^2^ = 0.47) but not IHC counts (*p* = 0.069, R^2^ = 0.117).

### Female mice are less susceptible to kanamycin-induced hearing loss

**Fig. 5:**
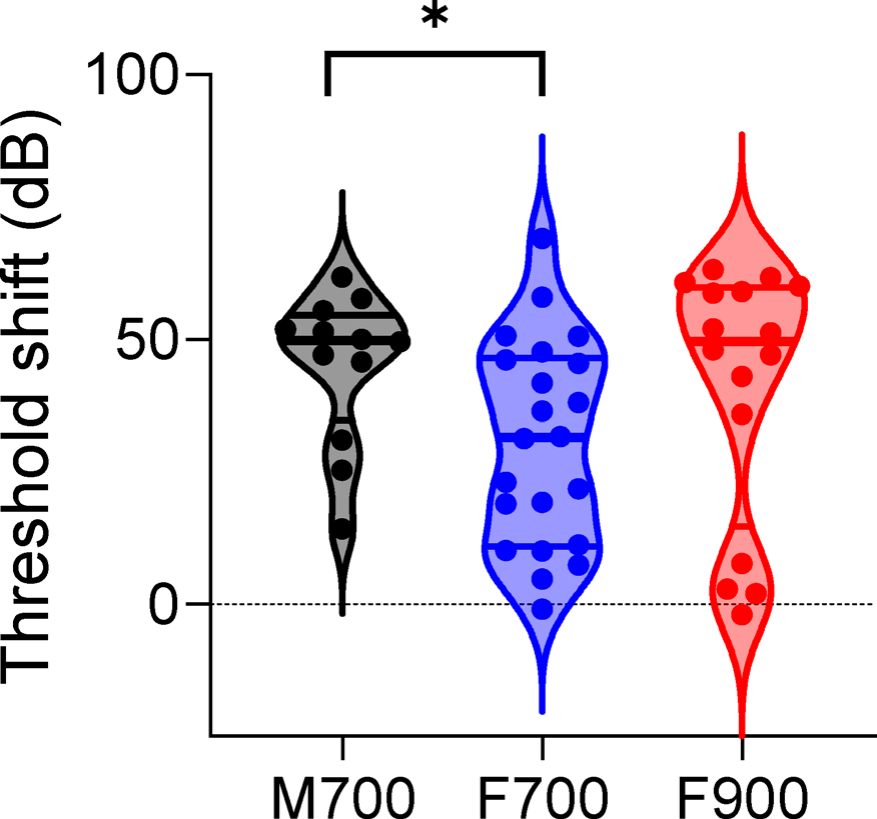
ABR threshold shifts in male and female mice treated with 700 or 900 mg/kg kanamycin. The threshold shifts (in dB) were calculated by subtracting baseline ABR thresholds from the ABR thresholds obtained 28 days (cohort 1) or 7 days (cohort 2) after deafening. M700: males treated with 700 mg/kg kanamycin (black); F700: females treated with 700 mg/kg kanamycin (blue); F900: females treated with 900 mg/kg kanamycin (red). The horizontal lines represent the median and the 25th and 75th percentiles.

We analyzed ABR threshold shifts after deafening relative to baseline in the two cohorts. We combined the ABR threshold shift data from cohort 1 (C57BL/6 J mice) and cohort 2 (Lgr5GFP transgenic mice). The ABR thresholds for the mice in cohort 2 were evaluated on days 0 and 7 only (Supplementary Fig. 1 and Supplementary Table 3). The threshold shifts of cohort 1 mice were determined on day 28 relative to day 0, and the threshold shifts of cohort 2 mice were determined on day 7 relative to day 0. According to our criterion of ototoxic response, a 40 dB threshold shift, we observed that in the M700 group, 3 out of the 12 males (25%) did not respond to the ototoxic insult, whereas 9 males responded (50 dB median, Fig. 5). In the F700 group, 14 out of 22 females (63%) did not respond to the ototoxic insult, and 8 females responded (32 dB median, Fig. 5). The differences in response rates between M700 and F700 were significant (*p* = 0.0031, χ^2^ = 4.63, two-sided chi-square test). The difference in the threshold shift between the M700 and F700 groups was significant (median values of 50 and 32 dB, respectively; *p* = 0.0205, Mann‒Whitney test; Fig. 5). Importantly, we observed no differences in ABR threshold shifts between cohort 1 and cohort 2 mice within the F700 group (medians 38 and 23, respectively, *p* = 0.40, Mann‒Whitney test). Note that the case where the threshold at day 28 was ≥ 105 dB pe SPL (no responses at the maximum click level), did not affect the statistical outcome as the threshold shift of this case had the highest rank. Thus, female mice treated with the same dose of kanamycin as males (700 mg/kg) in combination with furosemide are less likely to develop severe hearing loss.

We evaluated whether a higher dose of kanamycin (900 mg/kg, F900) would lead to an increase in the response of the females to the ototoxic insult. In the F900 group, 5 out of 16 females (31%) did not respond to the ototoxic insult, and 11 females responded (Fig. 5). There was a significant difference in the response rate between F700 and F900 (*p* = 0.049, χ^2^ = 3.89, chi-square test)**.** The differences in the threshold shifts between F900 and F700 were not significant (50 and 32 dB, respectively, *p* = 0.095, Mann‒Whitney test). Importantly, we observed no differences in ABR threshold shifts between cohort 1 and cohort 2 mice within the F900 group (medians 47 and 51, respectively, *p* = 0.41, Mann‒Whitney test). No significant differences were found in the response rates (*p* = 0.97, χ^2^ = 0.001, chi-square test) or ABR threshold shifts between F900 and M700 (medians 50 dB each, *p* = 0.92, Mann‒Whitney test; Fig. 5). These results suggest that a higher dose of kanamycin (900 mg/kg) results in a similar response rate in females as in males treated with the standard dose (700 mg/kg) of kanamycin.

## Discussion

The objective of this study was to investigate sex differences in the response to ototoxic medications, specifically kanamycin in C57BL/6 J mice. We evaluated the differences in ABR thresholds to click-stimuli as a measure of hearing performance between male and female C57BL/6 J mice exposed to kanamycin. Compared with male C57BL/6 J mice, female C57BL/6 J mice are more resistant to kanamycin-induced ototoxicity when the structural and functional outcomes of females and males treated with the same dose of kanamycin are compared. We found similar outcomes when females were treated with a substantially higher dose of kanamycin (900 vs 700 mg/kg) than males were. We propose the use of both male and female C57BL/6 J mice for kanamycin-induced ototoxicity models by treating females with a higher dose of kanamycin. The results of our study are consistent with those of previous studies showing that sex plays a role in susceptibility to hearing impairment^26–29^. With respect to diet-induced hearing impairment, female CBA/Ca mice are less prone to developing hearing loss than males^26^. Another study revealed that female B6CBAF1/J individuals develop less hearing impairment than males do after noise exposure^28^. Importantly, sex differences in the auditory function of mice, rats, Mongolian gerbils and chinchillas have been reported for normal-hearing models; mice and rats have been used for age-related hearing loss models; mice, rats, guinea pigs, Mongolian gerbils and chinchillas have been used for noise-induced hearing loss models; mice and rats have been used for cisplatin-induced ototoxicity models; and rats and guinea pigs have been used for ototoxic aminoglycoside models (reviewed previously 29). This finding shows that for preclinical studies in hearing, differences must be made in experimental approaches in male and female mice. Importantly, the sex differences in response to ototoxicity, hearing loss, and/or ageing are strain and species dependent. For example, DeBacker et al. showed that in cisplatin-induced hearing loss, male BALBc mice are more sensitive than females but in C57BL/6 J mice is the opposite with females being more sensitive^30^.

There is a lack of clinical data regarding sex differences in the response to ototoxicity in patients, and more importantly, contradictory results have been reported in different studies. For aminoglycoside antibiotics, one study reported that patients who developed ototoxicity after taking amikacin for the treatment of nontuberculous mycobacteria were more likely to be female^34^. The authors evaluated both subjective (self-reported) and objective hearing loss, and they reported that half of the patients with subjective symptoms of ototoxicity had normal audiograms, suggesting an important bias in the results. Compared with female patients, male patients have a greater risk of developing hearing loss^35–37^, which is consistent with the findings of preclinical research. Owing to the lack of meta-analyses, large cohort studies or systematic reviews on sex differences in response to different ototoxic agents, it is difficult to predict whether aminoglycoside antibiotics generate more hearing loss in men than in women, as seen in our study.

Estrogens have been associated with protection against hearing loss in patients and in different animal models of hearing loss^19–21^. It has been demonstrated that gonadectomy eliminates sex differences in response to noise exposure^28^ and that estrogen replacement therapy in females who have undergone gonadectomy protects them against noise damage^21^. In a study of premenopausal women aged 18–39 years, low hearing thresholds were observed during the late follicular phase of the menstrual cycle, corresponding to the highest levels of serum estrogen^38^.

For animal experiments on hearing loss, it is important to be aware of the sex differences that exist in response to age-related hearing loss, noise-induced hearing loss or ototoxicity. Nonetheless, it is imperative that we include female subjects in basic and preclinical research to improve the translation of therapies to the clinic. Here, we not only demonstrate a sex difference in response to kanamycin-induced ototoxicity in C57BL/6 J mice but also propose an experimental approach to induce deafening in female C57BL/6 J mice with a higher dose of kanamycin than that used for males. This will ensure that female C57BL/6 J mice are included in hearing loss research, help with the translation of basic research to the clinic, and reduce the number of animals used for this type of research (3Rs).

## Supplementary Information

Below is the link to the electronic supplementary material.

Supplementary Material 1

Supplementary Material 2

Supplementary Material 3

Supplementary Material 4

## Data Availability

The datasets acquired during the current study are available from the corresponding author upon reasonable request.
