# Supplementary material for: Sex differences in response to kanamycin-induced ototoxicity in C57BL/6 J mice
Source: Sci Rep. 2025 Oct 31;15:38139. doi: 10.1038/s41598-025-21962-y (PMC12579208; doi:10.1038/s41598-025-21962-y)
Supplement: Supplementary file 4 — Supplementary Material 4 [file 41598_2025_21962_MOESM4_ESM.docx]

Supplementary Figure 1


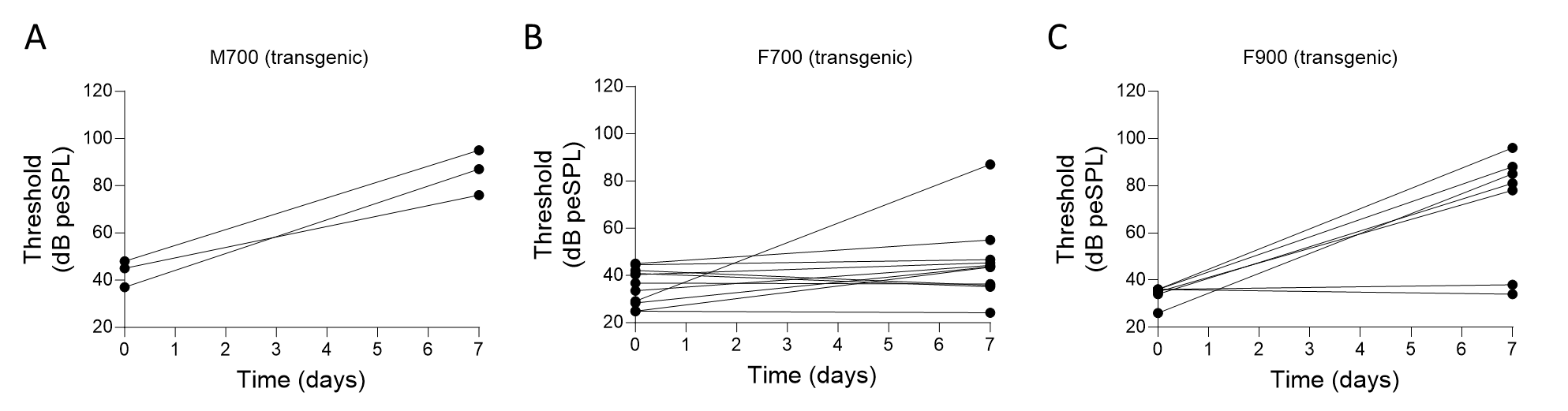


**Figure S1: ABR thresholds in male and female mice from cohort 2.** ABR thresholds (in dB peSPL) at days 0 and 7 in the group of male mice from cohort 2 that received a dose of 700 mg/kg kanamycin (M700), n=3 mice. B) ABR thresholds (in dB peSPL) at days 0 and 7 for the group of female mice from cohort 2 that received a dose of 700 mg/kg kanamycin (F700) (n=11 mice). C) ABR thresholds (in dB peSPL) at days 0 and 7 for the group of female mice from cohort 2 that received a dose of 900 mg/kg kanamycin (F900) (n=7 mice).
